# Evaluation of bovine coronavirus in Korean native calves challenged through different inoculation routes

**DOI:** 10.1186/s13567-024-01331-9

**Published:** 2024-06-11

**Authors:** Hyung-Chul Cho, Youngjun Kim, Yong-Il Cho, Jinho Park, Kyoung-Seong Choi

**Affiliations:** 1 Department of Animal Science and Biotechnology, College of Ecology and Environmental Science, Kyungpook National University, Sangju, 37224 Republic of Korea; 2 Department of Veterinary Internal Medicine, College of Veterinary Medicine, Jeonbuk University, Iksan, 54596 Republic of Korea; 3 Department of Animal Hospital, Hanwoo (Korean indigenous cattle) Genetic Improvement Center, National Agricultural Cooperative Federation, Seosan, 31948 Republic of Korea; 4 Department of Animal Science and Technology, College of Bio-Industry Science, Sunchon National University, Suncheon, 57922 Republic of Korea

**Keywords:** Bovine coronavirus, inoculation route, nasal discharge, virus shedding, interleukin-8

## Abstract

Bovine coronavirus (BCoV) is a pneumoenteric virus that can infect the digestive and respiratory tracts of cattle, resulting in economic losses. Despite its significance, information regarding BCoV pathogenesis is limited. Hence, we investigated clinical signs, patterns of viral shedding, changes in antibody abundance, and cytokine/chemokine production in calves inoculated with BCoV via intranasal and oral. Six clinically healthy Korean native calves (< 30 days old), initially negative for BCoV, were divided into intranasal and oral groups and monitored for 15 days post-infection (dpi). BCoV-infected calves exhibited clinical signs such as nasal discharge and diarrhea, starting at 3 dpi and recovering by 12 dpi, with nasal discharge being the most common symptoms. Viral RNA was detected in nasal and fecal samples from all infected calves. Nasal shedding occurred before fecal shedding regardless of the inoculation route; however, fecal shedding persisted longer. Although the number of partitions was very few, viral RNA was identified in the blood of two calves in the oral group at 7 dpi and 9 dpi using digital RT-PCR analysis. The effectiveness of maternal antibodies in preventing viral replication and shedding appeared limited. Our results showed interleukin (IL)-8 as the most common and highly induced chemokine. During BCoV infection, the levels of IL-8, monocyte chemoattractant protein-1, and macrophage inflammatory protein-1β were significantly affected, suggesting that these emerge as potential and reliable biomarkers for predicting BCoV infection. This study underscores the importance of BCoV as a major pathogen causing diarrhea and respiratory disease.

**Supplementary Information:**

The online version contains supplementary material available at 10.1186/s13567-024-01331-9.

## Introduction

Bovine coronavirus (BCoV) is a pneumoenteric virus that belongs to the species *Betacoronavirus* 1 (subgenus *Embecovirus*) and shared similarities with human coronavirus (HCoV)-OC43, which was originally transmitted from bovines [1]. BCoV causes diarrhea in calves, winter dysentery in adult cattle, and respiratory diseases in cattle of all ages [2], resulting in substantial economic losses due to decreased weight gain and milk production. Generally, BCoV infection causes profuse, watery diarrhea, with the feces possibly containing blood and mucus. The onset of clinical signs is noted approximately 2-days post-exposure and lasts for 3–6 days [3]. Research shows that BCoV detection in ≥ 70% of feces from clinically normal cows, with calves primarily infected through the fecal–oral route from their dam [4]. BCoV replicates in the epithelial cells of the colon crypts, destroying the villi and leading to degeneration, crypt epithelium necrosis, and petechial hemorrhage. This can result in severe hemorrhagic diarrhea in calves, which can be life-threatening because of electrolyte imbalance and malnutrition [5, 6].

Furthermore, BCoV has emerged as a major pathogen causing bovine respiratory disease (BRD), accompanied by pyrexia, nasal discharge, cough, or pneumonia in severe cases [7, 8]. Studies report BCoV prevalence ranging from 3.4 to 69% in diarrheal feces and 11.8–74.6% in BRD-associated symptoms [7, 9–11]. BCoV has also been reported to be 46% in calves with normal feces [12] and 63.4% in nasopharyngeal swabs of healthy cattle [7]. Although BCoV was first isolated from diarrheal calves and focused on enteric illness, recent studies have considered its association with respiratory diseases [13–16].

BCoVs have often been divided into either enteric or respiratory BCoVs based on the organ from which the virus was isolated. According to recent studies, BCoVs infections worldwide have dual respiratory and enteric tropisms, with the ability to infect both the respiratory and digestive tracts. However, a previous study performed by our group revealed no genetic or phylogenetic differences between enteric and respiratory BCoVs [17]. Therefore, there appeared to be no differences between respiratory and enteric BCoVs. Nevertheless, it remains unclear whether a single virus can infect multiple tissues or whether the virus infection site varies depending on tissue tropism [5].

Coronaviruses (CoVs) have broad tissue tropisms and sometimes cause systemic infections [18–20]. Some CoVs use monocytes/macrophages or lymphocytes for dissemination, suggesting that these viruses can circulate in the body via the bloodstream [20, 21]. Although several studies have confirmed that BCoV can infect various tissues in the respiratory and intestinal tracts, and even in the brain [22–24], only a few studies have reported how BCoV can spread to different organs within an infected animal [25]. However, because viremia is rarely detectable in BCoV infection, the mechanism by which the virus crosses the tissue and spreads to other organs remains unresolved [26].

Cytokines are not only solely involved in immune regulation but also have diverse physiological functions [27, 28]. Excessive or prolonged production of proinflammatory cytokines can lead to chronic inflammation, tissue damage, and autoimmune diseases [29, 30]. Conversely, inadequate or impaired cytokine production can result in immunodeficiency and heightened vulnerability to infections [31, 32]. Cytokines and chemokines are involved in immune and inflammatory responses against infectious diseases [33]. Previous studies have reported that HCoVs, including Middle East respiratory syndrome coronavirus (MERS-CoV) and severe acute respiratory syndrome coronavirus 2 (SARS-CoV-2), induce the increased production of proinflammatory cytokines, also known as a cytokine storm [34–36]. However, to date, there is limited information regarding BCoV infection and the production of cytokines/chemokine.

Moreover, data on BCoV infection and viral shedding are also scarce. A previous study reported that BCoV replication and shedding began on the 2nd day after exposure and that nasal secretion of the virus occurred sooner than that through feces [24]. On the other hand, studies have also shown that the first establishment of BCoV infection is dependent on the route of inoculation, with subsequent transmission of the virus to other tissues [37]. In this study, calves were infected with BCoV isolated from feces using two different inoculation routes. We monitored their clinical signs, compared the viral shedding through nasal and feces, assessed changes in antibody levels, and investigated cytokines/chemokines production.

## Materials and methods

### Virus preparation

Fecal samples (200 mg) from diarrheal calf that tested positive for BCoV by RT-PCR were subjected to virus isolation. Briefly, fecal suspensions were centrifuged at 2400 × *g* for 10 min, filtered using a 0.22-μm PES filter Corning^®^ Syringe Filter (Corning Inc., Corning, NY, USA), and inoculated on human rectal tumor-18G cells, which were grown in serum-free Dulbecco’s modified Eagle’s medium (Thermo Fisher Scientific, Waltham, MA, USA) supplemented with 0.1 mg/mL streptomycin, 100 U/mL penicillin (Thermo Fisher Scientific), and trypsin (5 μg/mL) (Thermo Fisher Scientific) for 4 days. Cytopathic effects were examined on days 2, 3, and 4 using inverted light microscopy. The cells were then frozen and thawed three times, and the supernatants were harvested for detection of BCoV by real-time RT-PCR targeting the nucleocapsid (N) gene. The Ct value for this virus was 12.73. Viral propagation and titration were performed using the Madin-Darby bovine kidney cell line as previously described [38].

### Experimental animals

Six Korean native calves, all younger than 30 days (mean age 23 ± 6 days), were selected for this study. These animals did not exhibit any clinical manifestations, including anorexia, coughing, depression, nasal discharges, or diarrhea. The calves were allocated into two groups for infection via different BCoV inoculation routes, each receiving 10^3.3^ TCID_50_/mL: intranasal (6 mL; 27 ± 5 days) and oral (5 mL; 18 ± 2 days). After inoculation, the calves were housed separately. Prior to the experiment, all calves were tested for BCoV antigen and results were negative. The dams of these animals were vaccinated against bovine rotavirus and BCoV and were fed colostrum after birth. BCoV antibody levels in the calves were also assessed 2 days before the experiment began, confirming all were seropositive.

### Sample collection and blood analysis

Following inoculation, the calves were monitored daily during the experiment and clinical signs were scored as mild, moderate, or severe. Nasal, rectal, and blood samples were collected at 1, 3, 5, 7, 9, 12, and 15 days post-infection (dpi). Additional nasal and rectal swabs were taken at 22 dpi to evaluate viral shedding. Blood was drawn from the jugular vein and divided into tubes containing EDTA (BD Vacutainer^®^, Becton Dickinson Vacutainer Systems, Franklin Lakes, NJ, USA) and into serum tubes (Vacutte^®^ serum tube, Greiner Bio-One, Austria). The collected samples were immediately transported to the laboratory on ice, and serum separation was performed for further analysis.

### BCoV detection by real-time RT-PCR

Viral RNA was extracted from nasal and rectal swabs using the AccuPrep^®^ Viral RNA Extraction Kit (Bioneer, Daejeon, Republic of Korea) and from blood using RNAiso (Takara, Shiga, Japan), as per the manufacturer’s guidelines. BCoV in nasal and rectal swabs, as well as in blood, was detected by real-time RT-PCR targeting the N gene [39, 45], and Ct values of ≤ 30 were considered positive. Each run included positive and negative controls. Viremia was assessed using both real-time and digital RT-PCR (RT-dPCR).

### Detection of BCoV in blood using RT-dPCR

RNA extracted from blood samples was subjected to RT-dPCR using the QIAcuity Digital PCR System (Qiagen, Hilden, Germany), according to the manufacturer’s instructions. The same primer sets utilized for real-time RT-PCR were employed. The RT-dPCR assays were conducted in a final reaction volume of 40 μL using the QIAcuity OneStep Advanced Probe Kit (Qiagen) containing 4 × OneStep Advanced Probe Master mix, 100 × OneStep RT mix, 400 nM of each primer, 200 nM of probe, PCR grade water, and RNA template in 26 K 24-well Nanoplate (Qiagen). Briefly, reverse transcription step was performed at 50 °C for 40 min, followed by an activation step at 95 °C for 2 min. The amplification consisted of 40 cycles at 95 °C for 5 s and 60 °C for 30 s. On average, 25 000 reactions were executed per sample. The QIAcuity Software Suite version 2.1.8 (Qiagen) was used to determine the sample thresholds using both positive and negative controls. The threshold was manually adjusted based on the positive control for each assay, and samples were deemed positive if they exceeded 0 copies/μL.

### Detection of BCoV antibodies using ELISA

Serum samples were evaluated for the detection of antibodies against BCoV using a commercially available indirect antibody enzyme linked immunosorbent assay (ELISA) kit (SVANOVIR^®^ BCV-Ab, Uppsala, Sweden) in accordance with the manufacturer’s instructions. The optical density (OD) was measured at 450 nm using an Infinite F50 microplate reader (Tecan, Mannedorf, Switzerland), which was used to calculate the BCoV antibody percent positivity (PP) by dividing the OD of the unknown sample by that of the positive control provided in the kit. A calculated PP value of > 10 was considered positive. The sensitivity of the kit is estimated at 84.6%, and its specificity is reported to be 100%.

### Cytokine/chemokine measurement

The concentration of each cytokine/chemokine in the serum was determined using Milliplex^®^ Bovine Cytokine/Chemokine Magnetic Bead Panel 1 (Millipore, Burlington, MA, USA) according to the manufacturer’s instructions. This panel included 15 different analytes: interferon-gamma (IFNγ), interleukin-1 alpha (IL)-1α, IL-1β, IL-4, IL-6, IL-8, IL-10, IL-17A, IL-36 receptor antagonist (IL-36RA), IFNγ inducible protein (IP-10, CXCL10), monocyte chemoattractant protein-1 (MCP-1, CCL2), macrophage inflammatory protein-1 alpha (MIP-1α, CCL3), MIP-1β (CCL4), tumor necrosis factor alpha (TNFα), and vascular endothelial growth factor (VEGF) A. The analytes were run on the Luminex^®^ analyzer (MAGPIX^®^) (Luminex Corp., Austin, TX, USA) and quantified using × POTENT^®^ software. The final concentrations of cytokines/chemokines (pg/mL) were determined by fitting to a standard curve using the five-parameter logistic method. All measured values were found to fall within the quality control ranges.

### Statistical analysis

Data are expressed as the mean ± standard deviation. Statistical analyses were performed using SPSS 29.0 software package (SPSS, Chicago, Illinois, USA). The variables of interest included cytokines/chemokines levels as dependent variables, with group and infection time at seven time points post-BCoV infection as independent variables. The effects of inoculation route and infection time, along with their interaction, were each analyzed using a two-way repeated measures analysis of variance (two-way RMANOVA). Additionally, differences among groups were assessed using a one-way RMANOVA. All ANOVA tests were followed by appropriate post-hoc multiple comparisons. Statistical significance was denoted as *P* < 0.05. All figures were processed using GraphPad Prism Version 8 (GraphPad Software, La Jolla, CA, USA).

## Results

### Clinical outcomes after BCoV exposure via two different routes

Among the six animals used in the experiment, five developed mild clinical signs such as diarrhea and nasal discharge. None of the infected calves showed signs of anorexia, coughing, or depression. As shown in Table 1, all calves infected by the intranasal inoculation exhibited nasal discharges, and two calves developed mild diarrhea and one calf developed moderate diarrhea; however, these symptoms recovered by 12 dpi. In contrast, all calves in the oral group had different clinical signs. One calf showed no clinical signs until the end of the experiment, whereas two exhibited nasal discharge and diarrhea, at 3 dpi (Table 1). A calf that exhibited nasal discharge did not display any clinical signs thereafter, whereas another calf with diarrhea developed nasal discharge at 9 dpi, and symptoms disappeared by 12 dpi (Table 1). Nasal discharge was the most common clinical sign observed, regardless of the inoculation route.

**Table 1:** **Scoring of clinical manifestations observed in BCoV-inoculated calves during this experiment.**

Inoculation route	Calves	Days post-infection							
		−2	1	3	5	7	9	12	15
Intranasal	1	–	–	–	–	D: + NS: +	D: + +	–	–
	2	–	–	D: + NS: +	–	–	–	–	–
	3	–	–	–	–	NS: +	–	–	–
Oral	1	–	–	–	–	–	–	–	–
	2	–	–	NS: +	–	–	–	–	–
	3	–	–	D: +	–	–	NS: +	–	–

D: diarrhea (loose to watery), NS: nasal discharge.

–: no sign, + : mild, +  + : moderate, +  +  + : severe.

### Nasal shedding of viral RNA

**Figure 1:**
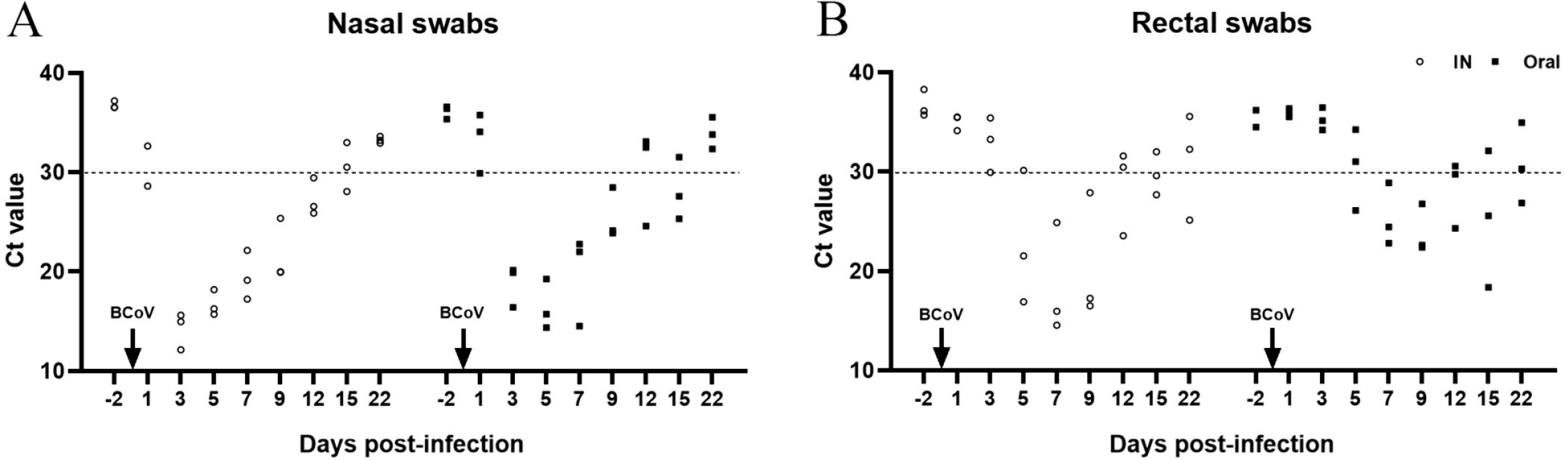
**Comparison of bovine coronavirus (BCoV) shedding patterns from nasal (A) and rectal (B) swabs between the IN (intranasal; white circle) and oral (black square) groups**. Real-time RT-PCR targeting the nucleocapsid gene was performed, and Ct values of ≤ 30 were considered positive.

The pattern of BCoV RNA shedding in nasal samples was compared according to the route of inoculation. Regardless of the inoculation route, virus shedding from nasal samples started at 1 dpi in one calf from each group, and the duration of shedding was different between the two groups (Figure 1A). In the intranasal group, viral RNA was detected from 3 to 12 dpi in all calves, and only one calf continuously shed BCoV until 15 dpi (Figure 1A). Meanwhile, in the oral group, BCoV RNA was present from 3 to 9 dpi, and was intermittently detected in one calf at 12 dpi and in two calves at 15 dpi (Figure 1A). None of nasal swabs from all calves were positive at 22 dpi (Figure 1A).

### Fecal shedding of viral RNA

Viral shedding in fecal samples varied based on the route of inoculation, and detection of viral RNA occurred later than in nasal swabs. At 3 dpi, BCoV was first detected in one calf from the intranasal group, but not in any calves from the oral group (Figure 1B). Two calves in the intranasal group were found to shed BCoV in their feces at 5 dpi, whereas in the oral group, only one calf was positive at this time point (Figure 1B). Between 7 and 9 dpi, all calves shed BCoV, regardless of the inoculation route (Figure 1B). Viral RNA was detected in one calf of the intranasal group and two calves of the oral group at 12 dpi. Viral RNA was intermittently detected until 22 dpi in one calf from each group (Figure 1B). BCoV shedding in feces tended to continue for a greater duration than that in nasal swabs.

### Association between clinical manifestation and viral shedding

The onset of clinical signs varied among individuals and was observed in three calves at 3 dpi. Nasal shedding of BCoV lasted for 6 days (from 3 to 9 dpi) in all calves; however, nasal discharge was not observed in these calves, and one calf exhibited no clinical signs during the experiment (Table 1). One calf in the oral group had mild diarrhea, but BCoV was not detected in the fecal samples of this calf. Nasal discharge, but not diarrhea, was a common clinical sign observed during BCoV infection. No clear correlation was found between clinical signs and viral shedding.

### Viral RNA in the blood samples

Using real-time RT-PCR, BCoV RNA was not detected in the blood samples from any of the calves until the end of the experiment. The follow-up analysis revealed that the RT-dPCR detection range for the N gene was 0.0–0.048 copies/μL. In the intranasal group, all samples were determined to be negative for BCoV, whereas in the oral group, two calves showed positive results (0.048 copies/μL) at 7 and 15 dpi for one calf, and at 9 dpi for the other. These values for the calves indicate that they are considered positive for BCoV, as noted in Table 2 and Additional file 1.

**Table 2:** **Amplification of BCoV RNAs in blood samples by digital RT-PCR.**

Inoculation routes	Calves	Days post-infection	Concentration	No. of partitions	Negative
				Positive	
Intranasal	1	1	0.000	0	25450
		3	0.000	0	25456
		5	0.000	0	25336
		7	0.000	0	25382
		9	0.000	0	25434
		12	0.000	0	25433
		15	0.000	0	25436
	2	1	0.000	0	23708
		3	0.000	0	25449
		5	0.000	0	25322
		7	0.000	0	25451
		9	0.000	0	25146
		12	0.000	0	25430
		15	0.000	0	25407
	3	1	0.000	0	25440
		3	0.000	0	25446
		5	0.000	0	25407
		7	0.000	0	25435
		9	0.000	0	23868
		12	0.000	0	25442
		15	0.000	0	25437
Oral	1	1	0.000	0	25438
		3	0.000	0	25408
		5	0.000	0	25380
		7	0.048	1	25387
		9	0.000	0	23947
		12	0.000	0	24785
		15	0.048	1	25453
	2	1	0.000	0	25448
		3	0.000	0	25453
		5	0.000	0	25480
		7	0.000	0	23364
		9	0.000	0	25473
		12	0.000	0	25217
		15	0.000	0	25463
	3	1	0.000	0	25371
		3	0.000	0	25478
		5	0.000	0	25439
		7	0.000	0	25418
		9	0.048	1	25451
		12	0.000	0	25448
		15	0.000	0	25403

### Antibody production after BCoV inoculation

**Figure 2:**
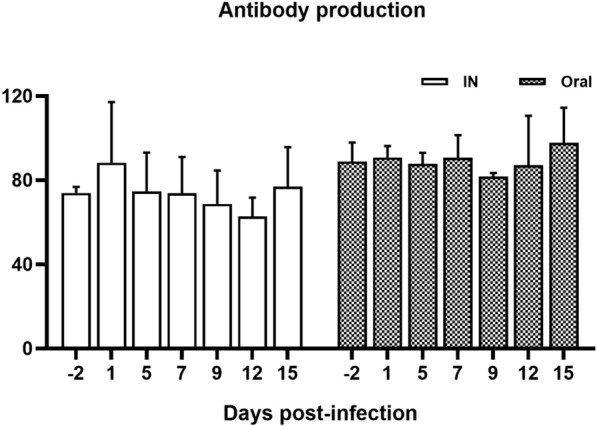
**Serum levels of anti-bovine coronavirus antibodies in calves infected through different inoculation routes**. Data are presented as the mean ± SD of each time point. The IN (intranasal) and oral groups are indicated by white and black boxes, respectively.

BCoV antibodies in serum were measured using ELISA. The antibody levels were higher in the oral group than in the intranasal group throughout the experiment (Figure 2). Nevertheless, there was no statistically significant difference between the groups (*P* = 0.167). In the intranasal group, the antibody levels were highest at 1 dpi, gradually decreased until 12 dpi, and then returned to similar to those before inoculation at 15 dpi (Figure 2). In contrast, BCoV antibody levels in the oral group generally remained high until 7 dpi, slightly decreased at 9 dpi, and then increased until 12 dpi, and the highest antibody production levels occurred at this point (Figure 2).

### Cytokine and chemokine levels in serum samples

**Figure 3:**
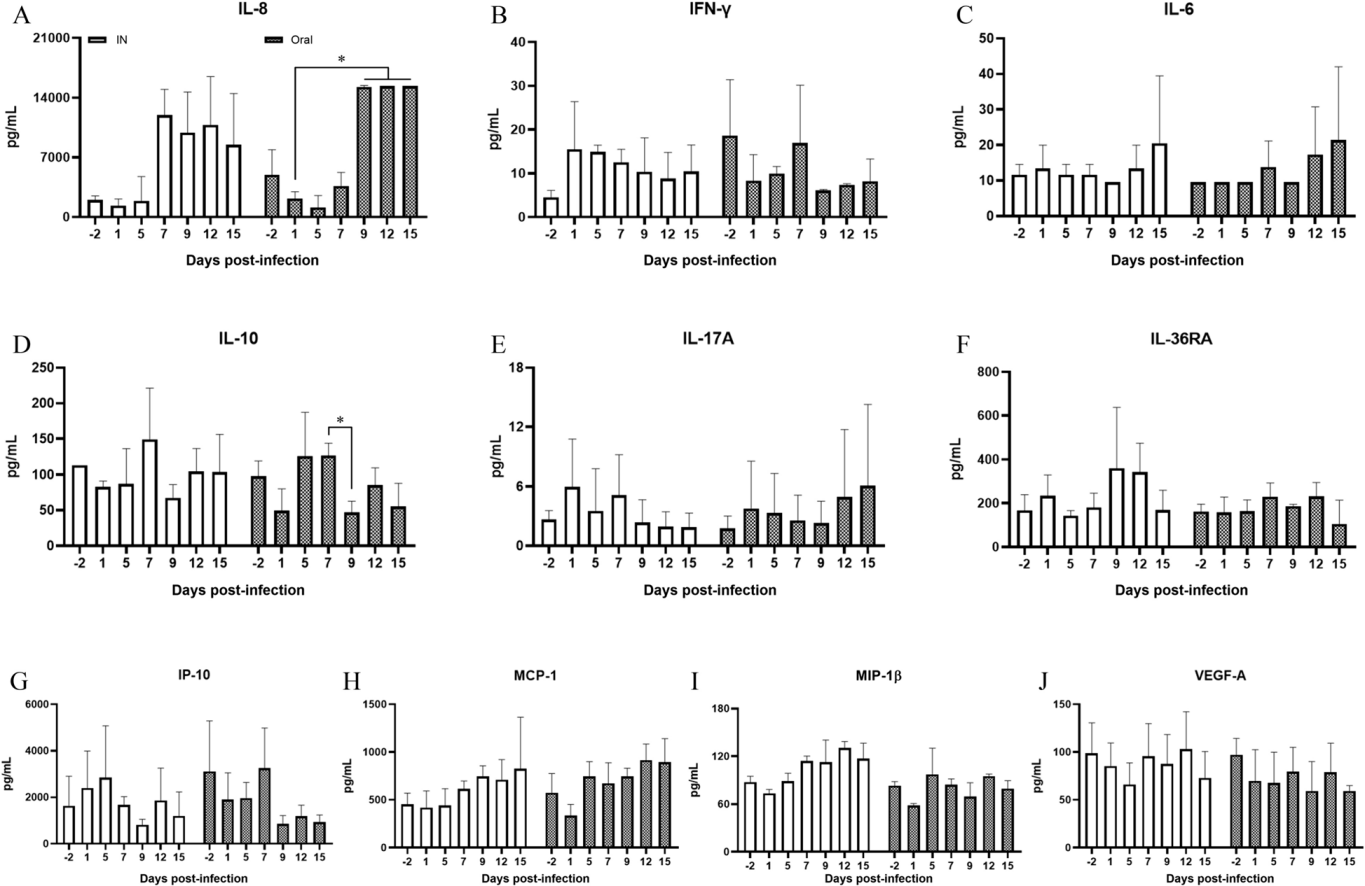
**Cytokine/chemokine concentrations of IL-8 (A), IFN-γ (B), IL-6 (C), IL-10 (D), IL-17A (E), IL-36RA (F), IP-10 (G), MCP-1 (H), MIP-1β (I), and VEGF-A (J) in the serum of calves after bovine coronavirus infection between the IN (intranasal; white box) and oral (black box) groups.** Data are presented as the mean ± SD of each time point. Statistical analyses were performed using two-way ANOVA with repeated measures. Significance levels are indicated by *p < 0.05, **p < 0.01, and *** p < 0.001.

Of the 15 analytes examined, IL-1α, IL-1β, IL-4, TNFα, and MIP-1α were undetectable, while IFNγ, IL-6, IL-10, IL-17A, IL-36RA, IL-8, IP-10, MCP-1, MIP-1β, and VEGF-A were detected in both groups (Figure 3). Two-way RMANOVA revealed no statistically significant differences between the intranasal and oral groups (Table 3). However, statistical significances were observed in the concentrations of IL-10 (*P* = 0.039), IL-8 (*P* = 0.000), MCP-1 (*P* = 0.014), MIP-1β (*P* = 0.044), and VEGF-A (*P* = 0.018), depending on the time of infection (Table 3). The two-way RMANOVA showed no interactions between group and time of infection (Table 3). Post-hoc analysis revealed that the concentration of IL-10 significantly decreased at day 9 dpi compared to day 7 dpi (*P* = 0.0273), and the concentration of IL-8 significantly increased at 9, 12, and 15 dpi compared to 1 dpi (Figure 3). Although MCP-1, MIP-1β, and VEGF-A demonstrated differences over time, there were not statistically significant in the post-hoc analysis. A one-way RMANOVA was conducted to assess differences between groups, revealing that IL-10 (*P* = 0.020), IL-8 (*P* = 0.006), and MCP-1 (*P* = 0.024) levels differed significantly in the oral group, whereas only MIP-1β showed a significant difference in the intranasal group (*P* = 0.005) (Additional file 2).

**Table 3:** **Results of the cytokine/chemokines levels after BCoV infection between the intranasal and oral groups.**

Factors	Two-way RMANOVA			Post-hoc analysis
	Infection time	Inoculation route	Interaction of variables	
IFN-γ	0.222	0.611	0.392	
IL-6	0.620	0.581	0.861	
IL-10	0.039^*^	0.235	0.584	0.020^*^
IL-17A	0.691	0.943	0.341	
IL-36RA	0.121	0.188	0.542	
IL-8	0.000^***^	0.166	0.659	0.006^**^
IP-10	0.154	0.491	0.584	
MCP-1	0.014^*^	0.167	0.501	0.024^*^
MIP-1β	0.044^*^	0.834	0.062	0.005^**^
VEGF-A	0.018^*^	0.517	0.712	

*p < 0.05, **p < 0.01, and ***p < 0.001.

IFNγ: interferon gamma, IL: interleukin, IL-36RA: IL-36 receptor antagonist, IP-10 (CXCL10): IFNγ inducible protein 10, MCP-1 (CCL2): monocyte chemoattractant protein-1, MIP-1β (CCL4): macrophage inflammatory protein-1 beta, VEFG-A: vascular endothelial growth factor A.

## Discussion

In this study, we investigated the clinical signs, viral shedding, changes in antibody abundance, and cytokine/chemokine concentrations in BCoV-infected calves exposed via different inoculation routes. Our results revealed that, regardless of the inoculation route, the onset of clinical symptoms varied among calves, starting at 3 dpi and lasting up to 9 dpi. Only two calves showed both mild nasal discharge and diarrhea; however, these symptoms did not persist for long. In particular, the clinical symptoms observed in BCoV-infected calves were generally mild, and most animals exhibited nasal discharge rather than diarrhea. This observation was in accordance with a previous study, which showed nasal discharge in all groups [24]. Because the virus used for inoculation was isolated from the fecal samples of a calf with diarrhea, we assumed that this virus would cause diarrhea in infected calves; however, contrary to our expectations, diarrhea was not observed in most calves. Furthermore, clinical symptoms were more noticeable in the intranasal group than in the oral group. The small sample size constrains our conclusions, yet the findings suggest that the primary manifestations in BCoV-infected calves might be nasal discharge. Several studies have reported that BCoV can cause respiratory symptoms, such as nasal discharge, sneezing, and coughing in calves aged 2–16 weeks [40–42] and may contribute to outbreaks of respiratory disease [39, 43, 44]. BCoV infections are also implicated in predisposing calves to severe secondary lower respiratory tract infections [40]. While the virulence of field strains of BCoV in causing respiratory tract diseases varies, the role of BCoV as a primary disease agent remains under debate [46]. However, our results suggest that BCoV predominantly affects the respiratory tract, not the gastrointestinal system, underscoring the need for further research to elucidate the link between BCoV infection and respiratory diseases.

We further compared the pattern of viral shedding between the nasal and feces according to the inoculation route. In the oral group, the pattern of virus shedding in the nasal secretions was similar to that of the intranasal group; however, BCoV shedding in the feces was detected slightly later in the oral group compared with that in the intranasal group, thus demonstrating the differences in the shedding patterns (Table 2). However, regardless of the inoculation route, the present results revealed that virus shedding from nasal swabs was observed 4 days earlier than that from feces in all calves, which was consistent with other studies, which revealed that nasal shedding preceded fecal shedding [24, 39]. Previous results showed that in intranasally inoculated calves, BCoV was first detected in the nasal discharge and then in the feces; however, when calves were orally inoculated, BCoV was first detected in feces and later in nasal discharge [37], suggesting that shedding patterns were dependent on the route of inoculation. Nonetheless, the findings from this study contrast with these earlier reports. The calves used in this study were tested for BCoV infection a week prior to the experiment, and it remains uncertain whether they had prior exposure to BCoV. Given that the calves intended for oral inoculation were 12 ± 2 days old at the start of the experiment, there is possibility that these animals were previously infected with BCoV and had developed diarrhea, potentially leading to delayed fecal shedding. However, there was no recorded history of intestinal diseases, such as diarrhea, among the calves used in this study. Therefore, our results suggest that virus shedding does not appear to be affected by the route of inoculation but may be more closely associated with the sequential continuity of infection (via the respiratory tract and then the gastrointestinal tract) of viral replication due to virus entry. In the natural environment, BCoV is transmitted via nasal secretions or the fecal–oral route [40]. After entering the host, the virus initially replicates in the upper respiratory tract, shielded by the mucous membrane. It then migrates through the nasal discharge to the gastrointestinal tract, resulting in a secondary infection that leads to intestinal replication and fecal shedding. These observations indicate that the respiratory pathway is a significant, and potentially dominant, route of BCoV infection under field conditions [24, 39, 41]. The pattern of shedding can be explained by the important role of BCoV as a pneumoenteric pathogen.

A recent study indicated that the nasal and fecal shedding periods lasted approximately 1–12 days and 2–17 days, respectively [24]. However, our results showed that in all BCoV-infected calves, the nasal shedding period was 3–9 days, whereas the fecal shedding period was 7–9 days, which was much shorter than previously reported results, especially in the feces [24, 42, 47]. The primary cause of this variation likely stems from the differences in the inoculation doses. Specifically, fecal shedding was shorter in the oral group, possibly because of the lower dose inoculated. Moreover, in this study, BCoV RNA in the nasal and fecal samples was intermittently detected up to 15 and 22 dpi, respectively. These findings are consistent with those of previous studies, in which viral shedding persisted longer in feces [24, 39]. This may be related to the viral transmission route, fecal–oral transmission. A notable limitation of this study is the absence of quantitative virus analysis in the nasal and fecal samples from the BCoV-infected calves. According to real-time RT-PCR analysis, Ct values, indicating viral presence, were generally lower (positivity) in nasal samples than in fecal samples, regardless of the route of inoculation. This suggests that the amount of virus shed differs between nasal secretions and feces following viral replication. However, the current data do not confirm whether the virus is active or infectious based on the obtained Ct value. Therefore, additional research is essential to quantify the viral load and ascertain the relationship between viral shedding and its transmissibility.

Previous studies have reported that nasal swab specimens appear to be more advantageous for virus detection in calves with suspected BCoV-associated diseases than fecal samples [24]. This is because, as shown in our results, viral RNA was more frequently positive in nasal swabs. In the intranasal group, viral RNA was consistently detected in nasal swabs but not in their feces. In contrast, the oral group showed a similar pattern of viral RNA detection in both nasal and fecal samples. Importantly, the sensitivity and specificity of these samples have not been assessed at this point; therefore, it is not possible to conclude which sample type is the most appropriate for diagnosis. However, considering the shedding pattern of the virus, it is deemed suitable to use both nasal and fecal samples simultaneously for BCoV diagnosis since detection time can vary depending on the sample.

Our results also showed no association between clinical signs and the detection of viral RNA. Several studies have reported that the virus is commonly detected in clinically healthy adults and calves [47–51], as well as in sick cattle. Furthermore, in this study, there was no direct correlation between the viral load (Ct value) and clinical symptoms, and the presence of BCoV RNA in animals does not necessarily mean that it is active or infectious [52]. In general, clinically healthy cattle can shed the virus via nasal secretions or feces for quite a long time [53], and it is assumed that when these cattle are subjected to environmental stresses, they may develop clinical signs and act as potential carriers. Given that, active surveillance of animals is required as the best strategy to control and prevent BCoV. Therefore, further studies should be aimed at establishing guidelines for viral load monitoring in cattle with the disease to provide exact information for the detection of BCoV in clinical samples.

Viral RNA was not detected in the blood using the real-time RT-PCR method, whereas it was identified in two calves of the oral group by RT-dPCR analysis between 7 and 15 dpi. Interestingly, one calf did not develop any clinical symptoms during the experiment, and the rest exhibited nasal discharge. These RT-PCR results were in accordance with those of a previous study [24] but were different from those reported by another study [25]. This discrepancy is because they used serum samples and nested PCR assays for BCoV detection [25]. In general, nested PCR is more sensitive than RT-qPCR but is also prone to contamination and more cumbersome [24, 47]. Although the viral load in the blood (the number of positive partitions) was low in this study, RT-dPCR results indicated that the oral route of infection could induce viremia. Previous studies have demonstrated that dPCR is a sensitive and accurate method for detecting low viral copy numbers [54]. Our findings suggest that RT-dPCR is appropriate for diagnosing viremia even when the viral RNA quantity is minimal. It remains uncertain whether the limited detection of viremia is due to the low virus dose used in inoculation or the low viral presence in the blood. We propose the latter scenario, as many viruses cause viremia, during which the virus circulates in the blood or blood cells and can disseminate to the target organs, initiating infection [55]. However, the mechanism by which BCoV reaches its target sites after infection is not well understood. One hypothesis is that only a portion of the virus circulates briefly in the blood during replication before entering the circulatory system. Consequently, highly sensitive detection methods are necessary for identifying BCoV. Similar to feline CoV, which utilizes monocyte-associated viremia for systemic spread [56]. BCoV might also move from the respiratory tract to the intestines or vice versa through a similar mechanism. Therefore, additional research is essential to elucidate how BCoV induces viremia.

Our findings revealed that the abundance of BCoV antibodies did not correlate with viral shedding. The ELISA test used in this study measured the total reactive rather than neutralizing antibodies. The dams of these calves were vaccinated before calving, and the calves therefore received passive immunity by ingesting colostrum, thereby obtaining antibodies against BCoV. Notably, nasal discharge rather than diarrhea was the primary symptom observed in these calves, despite their high antibody levels. This discrepancy may stem from variations in seroreactivity between the respiratory strains and the enteric reference strains used in the vaccines [44], potentially explaining the clinical signs observed in this study. Furthermore, considering the duration for which these calves were used in the experiment, the age of the calves in the intranasal group was approximately 42 ± 5 days (5–7 weeks), a time frame that might coincide with the declining levels of maternal antibodies, as opposed to the oral group, whose age was approximately 33 ± 2 days. We observed a more frequent alteration in BCoV antibody levels in the serum of the intranasal group, which correlated with onset of clinical symptoms. In contrast, calves in the oral group showed fewer clinical signs, suggesting that high levels of passive immunity could mitigate the severity or delay the onset of BCoV infection. The results indicate that passive immunity transferred from their dams would have protective effects against BCoV infection but a weak protective effect on viral replication. This is consistent with the results described in other studies [41, 57]. Although the amount of BCoV antibodies increased at 15 dpi in both groups compared with that before the experiment, viral shedding in the nasal and feces was still detected at this time point. Despite the high abundance of antibodies in serum, the amount of BCoV antibodies generated in this study may not be sufficient to contend with virus replication and shedding. Additionally, this may be because of a lack of a cell-mediated immune response to BCoV infection. Currently, data on T-cell immunity in relation to BCoV infection are scant [58]. Thus, further studies are necessary to elucidate the appropriate immune response that can prevent viral replication, shedding, and transmission during BCoV infection.

Among the cytokines and chemokines examined in this study, a significant increase in IL-8 was observed exclusively in the oral group (*P* = 0.006), with levels notably rising from 9 dpi until the conclusion of the experiment. IL-8 levels are similarly elevated in patients infected with SARS-CoV-2 [59–61]. The mechanism behind the elevated IL-8 levels in BCoV-infected calve remains unclear. In cases of SARS-CoV-2 infection, a surge in proinflammatory cytokines, including IL-1, IL-6, TNFα, and IFNγ, along with IL-8, is considered a major contributor to mortality [62, 63]. Conversely, in BCoV-infected calves, IL-1 and TNFα were undetectable, and IL-6 and IFNγ were present only in low quantities, with no significant variances observed. This could be attributed to the administration of a low BCoV dose used for inoculation, which did not cause a systemic infection in these animals. Furthermore, our findings indicate a statistically significant change in IL-10 levels in the oral group (*P* = 0.020), which displayed a marked decrease at 9 dpi relative to 7 dpi. The temporal increase in IL-10 may be linked to a regulatory effect by IL-8. Another noteworthy observation was the declining trend in VEGF-A levels over time in the oral group. Although changes in VEGF-A were not statistically significant in one-way RMANOVA, two-way RMANOVA results revealed a consistent decrease associated with the duration of infection after oral inoculation. VEGF-A is known for its varied biological roles, particularly in promoting angiogenesis. Several studies have shown that VEGF-A levels increases during viral infections [64–66]. However, our results were inconsistent with those of other studies, suggesting that BCoV infection may not trigger immune activation as indicated by the absence of proinflammatory cytokines induction. Notably, the duration of infection in calves significantly affected the levels of MCP-1 and MIP-1β. MCP-1, a chemokine that attracts monocytes and dendritic cells to infection sites [67, 68] was elevated in the oral group and consistent with that observed in patients with SARS-CoV-2 [69]. This elevation may be linked to an increase in monocytes after BCoV infection, a phenomenon supported by several studies [3, 7]. Although hematological results were not presented, our data indicated monocytosis in BCoV-infected calves (data not published). Furthermore, Alhetheel et al. reported that MCP-1 is the only chemokine associated with increased mortality risk in MERS-CoV infections [70]. MIP-1β elevation occurred in the intranasal group and is known for its role in recruiting and activating monocytes, lymphocytes, and natural killer cells [71]. This increase might also relate to monocytosis. Our data suggest that the chemokine profiles in BCoV-infected calves vary with the route of inoculation, particularly in the oral group. The similar patterns of IL-8, MCP-1, and MIP-1β elevation observed in both BCoV-infected calves and SARS-CoV-2 patients highlight this finding [72]. Given the lack of supporting data on the link between BCoV infection and cytokine/chemokine responses, additional research is required to clarify the immune mechanisms involved in BCoV infection.

The primary limitations of this study stem from the different volumes used for inoculation and the varying ages of calves across the two groups. It is challenging to determine whether these variations influenced the clinical signs and fecal shedding observed in the orally inoculated group, yet their potential impact cannot be dismissed. Although the inoculation dose and animal age are crucial factors in in vivo experiments, they seemingly did not substantially affect the outcomes of this study. Notably, the reasons for the significant alterations in cytokine/chemokine levels in the oral group remain unexplained. Despite these limitations, our results contribute important insights into the pathogenesis of BCoV.

In conclusion, this study demonstrated that nasal shedding of the virus preceded fecal shedding, and viral shedding persisted longer in feces than in nasal swabs, regardless of the inoculation route. Given that nasal discharge is a frequent symptom in BCoV-infected calves, these findings highlight the role of BCoV as a respiratory pathogen. Based on the shedding patterns observed, it is advisable to concurrently collect both nasal and fecal samples for accurate BCoV diagnosis. We found no direct correlation between clinical signs and the extent of virus shedding. Additionally, passive antibodies from the dam appeared to offer limited protection against viral replication. These results indicate that serum levels of IL-8, MCP-1, and MIP-1β may serve as viable and reliable biomarkers for predicting BCoV infection.

### Supplementary Information

**Additional file 1.** **Detection of bovine coronavirus RNA in the blood by digital RT-PCR. Each well contained an average of 25 000 partitions.** The sample threshold was determined using the positive and negative control wells in each test by applying the manual global threshold approach, which is based on the signal amplitude observed in negative control samples. The red line represents the test threshold, and a dot (one fluorescent partition) above the threshold is considered a positive result.  

**Additional file 2. Statistical results of IL-10, IL-8, MCP-1, and MIP-1β by one-way repeated measures analysis of variance.**

## Data Availability

The data supporting the findings of this study are available by the corresponding author upon reasonable request.

## References

[1] Jevsnik Virant M, Cerne D, Petrovec M, Paller T, Toplak I (2021). Genetic characterisation and comparison of three human coronaviruses (HKU1, OC43, 229E) from patients and bovine coronavirus (BCoV) from cattle with respiratory disease in Slovenia. Viruses.

[2] Carman PS, Hazlett MJ (1992). Bovine coronavirus infection in Ontario 1990–1991. Can Vet J.

[3] Chae JB, Park J, Jung SH, Kang JH, Chae JS, Choi KS (2019). Acute phase response in bovine coronavirus positive post-weaned calves with diarrhea. Acta Vet Scand.

[4] Crouch CF, Acres SD (1984). Prevalence of rotavirus and coronavirus antigens in the feces of normal cows. Can J Comp Med.

[5] Boileau MJ, Kapil S (2010). Bovine coronavirus associated syndromes. Vet Clin North Am Food Anim Pract.

[6] Gunn L, Collins PJ, O'Connell MJ, O'Shea H (2015). Phylogenetic investigation of enteric bovine coronavirus in Ireland reveals partitioning between European and global strains. Ir Vet J.

[7] Pratelli A, Cirone F, Capozza P, Trotta A, Corrente M, Balestrieri A, Buonavoglia C (2021). Bovine respiratory disease in beef calves supported long transport stress: an epidemiological study and strategies for control and prevention. Res Vet Sci.

[8] Vlasova AN, Saif LJ (2021). Bovine coronavirus and the associated diseases. Front Vet Sci.

[9] Pardon B, Callens J, Maris J, Allais L, Van Praet W, Deprez P, Ribbens S (2020). Pathogen-specific risk factors in acute outbreaks of respiratory disease in calves. J Dairy Sci.

[10] Fanelli A, Cirilli M, Lucente MS, Zarea AAK, Buonavoglia D, Tempesta M, Greco G (2021). Fatal calf pneumonia outbreaks in Italian dairy herds involving *Mycoplasma bovis* and other agents of BRD complex. Front Vet Sci.

[11] Calderon Bernal JM, Fernandez A, Arnal JL, Baselga C, Benito Zuniga A, Fernandez-Garyzabal JF, Vela Alonso AI, Cid D (2023). Cluster analysis of bovine respiratory disease (BRD)-associated pathogens shows the existence of two epidemiological patterns in BRD outbreaks. Vet Microbiol.

[12] Zhu Q, Su M, Li Z, Wang X, Qi S, Zhao F, Li L, Guo D, Feng L, Li B, Sun D (2022). Epidemiological survey and genetic diversity of bovine coronavirus in Northeast China. Virus Res.

[13] Mebus CA, Underdahl NR, Twiehaus MJ (1972). Isolation unit used in studies on neonatal calf diarrhea. Am J Vet Res.

[14] Thomas LH, Gourlay RN, Stott EJ, Howard CJ, Bridger JC (1982). A search for new microorganisms in calf pneumonia by the inoculation of gnotobiotic calves. Res Vet Sci.

[15] Zhu Q, Li B, Sun D (2022). Advances in bovine coronavirus epidemiology. Viruses.

[16] Geng HL, Meng XZ, Yan WL, Li XM, Jiang J, Ni HB, Liu WH (2023). Prevalence of bovine coronavirus in cattle in China: a systematic review and meta-analysis. Microb Pathog.

[17] Kim EM, Cho HC, Shin SU, Park J, Choi KS (2022). Prevalence and genetic characterization of bovine coronavirus identified from diarrheic pre-weaned native Korean calves from 2019 to 2021. Infect Genet Evol.

[18] Holmes KV (1999). Coronaviruses (Coronaviridae). Encyclopedia Virol.

[19] Decaro N, Buonavoglia C (2011). Canine coronavirus: not only an enteric pathogen. Vet Clin North Am Small Anim Pract.

[20] Porter E, Tasker S, Day MJ, Harley R, Kipar A, Siddell SG, Helps CR (2014). Amino acid changes in the spike protein of feline coronavirus correlate with systemic spread of virus from the intestine and not with feline infectious peritonitis. Vet Res.

[21] Marinaro M, Mari V, Bellacicco AL, Tarsitano E, Elia G, Losurdo M, Rezza G, Buonavoglia C, Decaro N (2010). Prolonged depletion of circulating CD4+ T lymphocytes and acute monocytosis after pantropic canine coronavirus infection in dogs. Virus Res.

[22] Kapil S, Trent AM, Goyal SM (1990). Excretion and persistence of bovine coronavirus in neonatal calves. Arch Virol.

[23] Hansa A, Rai R, Wani MY, Dhama K (2012). ELISA and RT-PCR based detection of bovine coronavirus in northern India. Asian J Anim Vet Adv.

[24] Oma VS, Traven M, Alenius S, Myrmel M, Stokstad M (2016). Bovine coronavirus in naturally and experimentally exposed calves; viral shedding and the potential for transmission. Virol J.

[25] Park SJ, Lim GK, Park SI, Kim HH, Koh HB, Cho KO (2007). Detection and molecular characterization of calf diarrhoea bovine coronaviruses circulating in South Korea during 2004–2005. Zoonoses Public Health.

[26] Park SJ, Kim GY, Choy HE, Hong YJ, Saif LJ, Jeong JH, Park SI, Kim HH, Kim SK, Shin SS, Kang MI, Cho KO (2007). Dual enteric and respiratory tropisms of winter dysentery bovine coronavirus in calves. Arch Virol.

[27] Kao CY, Mills JA, Burke CJ, Morse B, Marques BF (2023). Role of cytokines and growth factors in the manufacturing of iPSC-derived allogeneic cell therapy products. Biology.

[28] Xiao T, Yan Z, Xiao S, Xia Y (2020). Proinflammatory cytokines regulate epidermal stem cells in wound epithelialization. Stem Cell Res Ther.

[29] McInnes IB, Schett G (2007). Cytokines in the pathogenesis of rheumatoid arthritis. Nat Rev Immunol.

[30] Chen L, Kuang P, Liu H, Wei Q, Cui H, Fang J, Zuo Z, Deng J, Li Y, Wang X, Zhao L (2019). Sodium fluoride (NaF) induces inflammatory responses via activating MAPKs/NF-kappaB signaling pathway and reducing anti-inflammatory cytokine expression in the mouse liver. Biol Trace Elem Res.

[31] Bustamante J, Boisson-Dupuis S, Abel L, Casanova JL (2014). Mendelian susceptibility to mycobacterial disease: genetic, immunological, and clinical features of inborn errors of IFN-gamma immunity. Semin Immunol.

[32] Carvalho NB, de Lourdes BM, Souza AS, Netto EM, Arruda S, Santos SB, Carvalho EM (2018). Impaired TNF, IL-1beta, and IL-17 production and increased susceptibility to *Mycobacterium tuberculosis* infection in HTLV-1 infected individuals. Tuberculosis.

[33] Arai K, Nishida J, Hayashida K, Hatake K, Kitamura T, Miyajima A, Arai N, Yokota T (1990). Coordinate regulation of immune and inflammatory responses by cytokines. Rinsho Byori.

[34] Lau SKP, Lau CCY, Chan KH, Li CPY, Chen H, Jin DY, Chan JFW, Woo PCY, Yuen KY (2013). Delayed induction of proinflammatory cytokines and suppression of innate antiviral response by the novel Middle East respiratory syndrome coronavirus: implications for pathogenesis and treatment. J Gen Virol.

[35] Fung SJ, Joshi D, Fillman SG, Weickert CS (2014). High white matter neuron density with elevated cortical cytokine expression in schizophrenia. Biol Psychiatry.

[36] Gao YM, Xu G, Wang B, Liu BC (2021). Cytokine storm syndrome in coronavirus disease 2019: a narrative review. J Intern Med.

[37] Saif LJ, Redman DR, Moorhead PD, Theil KW (1986). Experimentally induced coronavirus infections in calves: viral replication in the respiratory and intestinal tracts. Am J Vet Res.

[38] Zhukhovitsky V, Shevlyagina N, Zubasheva M, Russu L, Gushchin V, Meerovich G, Strakhovskaya M (2022). Infectivity and morphology of bovine coronavirus inactivated in vitro by cationic photosensitizers. Viruses.

[39] Cho YI, Kim  WI, Kinyon JM, Yoon  KJ (2010). Development of a panel of multiplex real-time polymerase chain reaction assays for simultaneous detection of major agents causing calf diarrhea in feces. J Vet Diagn Invest.

[40] Clark MA (1993). Bovine coronavirus. Br Vet J.

[41] Heckert RA, Saif LJ, Mengel JP, Myers GW (1991). Mucosal and systemic antibody responses to bovine coronavirus structural proteins in experimentally challenge-exposed calves fed low or high amounts of colostral antibodies. Am J Vet Res.

[42] Saif LJ (2010). Bovine respiratory coronavirus. Vet Clin North Am Food Anim Pract.

[43] Hick PM, Read AJ, Lugton I, Busfield F, Dawood KE, Gabor L, Hornitzky M, Kirkland PD (2012). Coronavirus infection in intensively managed cattle with respiratory disease. Aust Vet J.

[44] Workman AM, Kuehn LA, McDaneld TG, Clawson ML, Chitko-McKown CG, Loy JD (2017). Evaluation of the effect of serum antibody abundance against bovine coronavirus on bovine coronavirus shedding and risk of respiratory tract disease in beef calves from birth through the first five weeks in a feedlot. Am J Vet Res.

[45] Thomas CJ, Hoet AE, Sreevatsan S, Wittum TE, Briggs RE, Duff GC, Saif LJ (2006) Transmission of bovine coronavirus and serologic responses in feedlot calves under field conditions. Am J Vet Res 67:1412-1420 10.2460/ajvr.67.8.1412 16881855

[46] McNulty MS, Bryson DG, Allan GM, Logan EF (1984). Coronavirus infection of the bovine respiratory tract. Vet Microbiol.

[47] Cho KO, Hasoksuz M, Nielsen PR, Chang KO, Lathrop S, Saif LJ (2001). Cross-protection studies between respiratory and calf diarrhea and winter dysentery coronavirus strains in calves and RT-PCR and nested PCR for their detection. Arch Virol.

[48] Crouch CF, Bielefeldt Ohmann H, Watts TC, Babiuk LA (1985). Chronic shedding of bovine enteric coronavirus antigen-antibody complexes by clinically normal cows. J Gen Virol.

[49] Bartels CJ, Holzhauer M, Jorritsma R, Swart WA, Lam TJ (2010). Prevalence, prediction and risk factors of enteropathogens in normal and non-normal faeces of young Dutch dairy calves. Prev Vet Med.

[50] Coura FM, Freitas MD, Ribeiro J, de Leme RA, de Souza C, Alfieri AA, Facury Filho EJ, de Carvalho AU, Silva MX, Lage AP, Heinemann MB (2015). Longitudinal study of *Salmonella* spp., diarrheagenic *Escherichia coli*, rotavirus, and coronavirus isolated from healthy and diarrheic calves in a Brazilian dairy herd. Trop Anim Health Prod.

[51] Ryu JH, Shin SU, Choi KS (2020). Molecular surveillance of viral pathogens associated with diarrhea in pre-weaned Korean native calves. Trop Anim Health Prod.

[52] Ellis J (2019). What is the evidence that bovine coronavirus is a biologically significant respiratory pathogen in cattle?. Can Vet J.

[53] Kanno T, Ishihara R, Hatama S, Uchida I (2018). A long-term animal experiment indicating persistent infection of bovine coronavirus in cattle. J Vet Med Sci.

[54] Park C, Lee J, Hassan ZU, Ku KB, Kim SJ, Kim HG, Park EC, Park GS, Park D, Baek SH, Park D, Lee J, Jeon S, Kim S, Lee CS, Yoo HM, Kim S (2021). Comparison of digital PCR and quantitative PCR with various SARS-CoV-2 primer-probe sets. J Microbiol Biotechnol.

[55] Shi X, Gong E, Gao D, Zhang B, Zheng J, Gao Z, Zhong Y, Zou W, Wu B, Fang W, Liao S, Wang S, Xie Z, Lu M, Hou L, Zhong H, Shao H, Li N, Liu C, Pei F, Yang J, Wang Y, Han Z, Shi X, Zhang Q, You J, Zhu X, Gu J (2005). Severe acute respiratory syndrome associated coronavirus is detected in intestinal tissues of fatal cases. Am J Gastroenterol.

[56] Kipar A, Baptiste K, Barth A, Reinacher M (2006). Natural FCoV infection: cats with FIP exhibit significantly higher viral loads than healthy infected cats. J Feline Med Surg.

[57] Lathrop SL, Wittum TE, Loerch SC, Perino LJ, Saif LJ (2000). Antibody titers against bovine coronavirus and shedding of the virus via the respiratory tract in feedlot cattle. Am J Vet Res.

[58] Kapil S, Goyal SM, Trent AM (1994). Cellular immune status of coronavirus-infected neonatal calves. Comp Immunol Microbiol Infect Dis.

[59] Cabaro S, D'Esposito V, Di Matola T, Sale S, Cennamo M, Terracciano D, Parisi V, Oriente F, Portella G, Beguinot F, Atripaldi L, Sansone M, Formisano P (2021). Cytokine signature and COVID-19 prediction models in the two waves of pandemics. Sci Rep.

[60] Kesmez Can F, Ozkurt Z, Ozturk N, Sezen S (2021). Effect of IL-6, IL-8/CXCL8, IP-10/CXCL 10 levels on the severity in COVID 19 infection. Int J Clin Pract.

[61] Kleymenov DA, Bykonia EN, Popova LI, Mazunina EP, Gushchin VA, Kolobukhina LV, Burgasova OA, Kruzhkova IS, Kuznetsova NA, Shidlovskaya EV, Divisenko EV, Pochtovyi AA, Bacalin VV, Smetanina SV, Tkachuk AP, Logunov DY, Gintsburg AL (2021). A deep look into COVID-19 severity through dynamic changes in blood cytokine levels. Front Immunol.

[62] Gonzalez-Rubio J, Navarro-Lopez C, Lopez-Najera E, Lopez-Najera A, Jimenez-Diaz L, Navarro-Lopez JD, Najera A (2020). Cytokine release syndrome (CRS) and nicotine in COVID-19 patients: trying to calm the storm. Front Immunol.

[63] Hsu RJ, Yu WC, Peng GR, Ye CH, Hu S, Chong PCT, Yap KY, Lee JYC, Lin WC, Yu SH (2022). The role of cytokines and chemokines in severe acute respiratory syndrome coronavirus 2 infections. Front Immunol.

[64] Spiropoulou CF, Srikiatkhachorn A (2013). The role of endothelial activation in dengue hemorrhagic fever and hantavirus pulmonary syndrome. Virulence.

[65] Kovacs-Kasa A, Zaied AA, Leanhart S, Koseoglu M, Sridhar S, Lucas R, Fulton DJ, Vazquez JA, Annex BH (2022). Elevated cytokine levels in plasma of patients with SARS-CoV-2 do not contribute to pulmonary microvascular endothelial permeability. Microbiol Spectr.

[66] Mukherjee S, Saha B, Tripathi A (2022). Clinical significance of differential serum-signatures for early prediction of severe dengue among Eastern Indian patients. Clin Exp Immunol.

[67] Deshmane SL, Kremlev S, Amini S, Sawaya BE (2009). Monocyte chemoattractant protein-1 (MCP-1): an overview. J Interferon Cytokine Res.

[68] Singh S, Anshita D, Ravichandiran V (2021). MCP-1: function, regulation, and involvement in disease. Int Immunopharmacol.

[69] Eichhorn T, Huber S, Weiss R, Ebeyer-Masotta M, Laukova L, Emprechtinger R, Bellmann-Weiler R, Lorenz I, Martini J, Pirklbauer M, Orth-Holler D, Wurzner R, Weber V (2023). Infection with SARS-CoV-2 is associated with elevated levels of IP-10, MCP-1, and IL-13 in sepsis patients. Diagnostics.

[70] Alhetheel A, Albarrag A, Shakoor Z, Somily A, Barry M, Altalhi H, Bakhrebah M, Nassar M, Alfageeh M, Assiri A, Alfaraj S, Memish Z (2023). Chemokine levels among patients with Middle East respiratory syndrome coronavirus infection. Vaccines.

[71] Chaisavaneeyakorn S, Moore JM, Mirel L, Othoro C, Otieno J, Chaiyaroj SC, Shi YP, Nahlen BL, Lal AA, Udhayakumar V (2003). Levels of macrophage inflammatory protein 1 alpha (MIP-1 alpha) and MIP-1 beta in intervillous blood plasma samples from women with placental malaria and human immunodeficiency virus infection. Clin Diagn Lab Immunol.

[72] Yadav PD, Sahay RR, Salwe S, Trimbake D, Babar P, Sapkal GN, Deshpande GR, Bhise K, Shete AM, Abraham P, Tripathy AS (2023). Broadly reactive SARS-CoV-2-specific T-cell response and participation of memory B and T cells in patients with Omicron COVID-19 infection. J Immunol Res.

