# Supplementary figures and images for: Evaluation of bovine coronavirus in Korean native calves challenged through different inoculation routes
Source: Vet Res. 2024 Jun 11;55:74. doi: 10.1186/s13567-024-01331-9 (PMC11165853; doi:10.1186/s13567-024-01331-9)

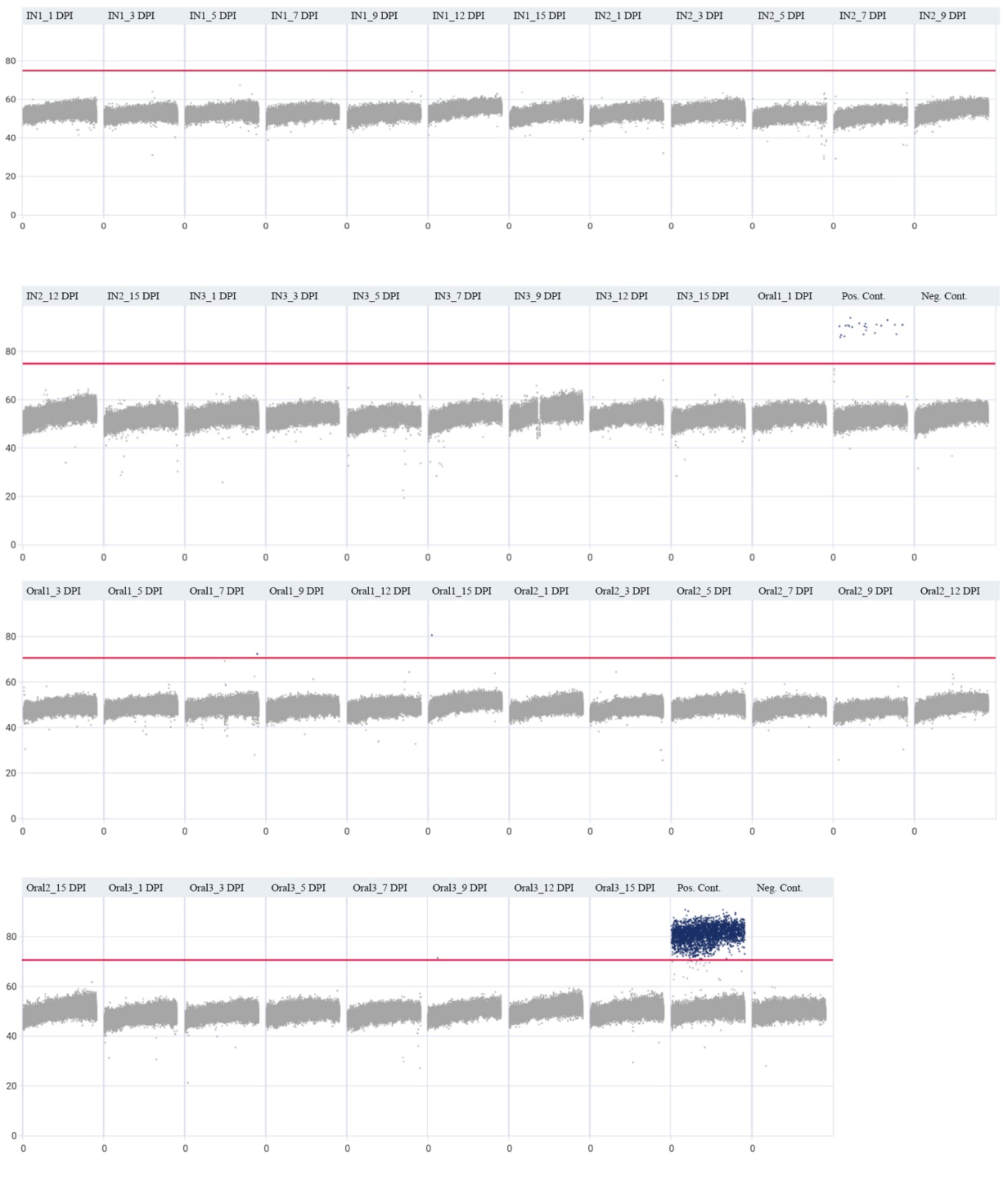

Supplement: Supplementary file 1 — Additional file 1. Detection of bovine coronavirus RNA in the blood by digital RT-PCR. Each well contained an average of 25 000 partitions. The sample threshold was determined using the positive and negative control wells in each test by applying the manual global threshold approach, which is based on the signal amplitude observed in negative control samples. The red line represents the test threshold, and a dot (one fluorescent partition) above the threshold is considered a positive result. [file 13567_2024_1331_MOESM1_ESM.docx]
