# Supplementary material for: Evaluation of bovine coronavirus in Korean native calves challenged through different inoculation routes
Source: Vet Res. 2024 Jun 11;55:74. doi: 10.1186/s13567-024-01331-9 (PMC11165853; doi:10.1186/s13567-024-01331-9)
Supplement: Supplementary file 2 — Additional file 2. Statistical results of IL-10, IL-8, MCP-1, and MIP-1β by one-way repeated measures analysis of variance. [file 13567_2024_1331_MOESM2_ESM.docx]

**Additional file 2. Statistical results of IL-10, IL-8, MCP-1, and MIP-1β by one-way repeated measures analysis of variance**

|  | Days post-infection | | Mean | SD | SE | 95% CI | *P*-value |  |
| --- | --- | --- | --- | --- | --- | --- | --- | --- |
| IL-10 (oral) | −2 | | 98.11 | 21.23 | 12.26 | 45.37 − 150.85 | 0.020 |  |
|  | 1 | | 49.63 | 30.53 | 17.62 | −26.20 − 125.47 |  |  |
|  | 5 | | 125.93 | 61.54 | 35.53 | −26.95 − 278.82 |  |  |
|  | 7 | | 126.7 | 17.13 | 9.89 | 84.16 − 169.24 |  |  |
|  | 9 | | 46.96 | 15.89 | 9.18 | 7.44 − 86.44 |  |  |
|  | 12 | | 85.47 | 24.12 | 13.92 | 25.56 − 145.38 |  |  |
|  | 15 | | 55.25 | 32.86 | 18.97 | −26.39 − 136.89 |  |  |
| IL-8 (oral) | −2 | | 4946 | 2952.58 | 1704.67 | −2388.69 − 12280.69 | 0.006 |  |
|  | 1 | | 2156 | 808.21 | 466.62 | 148.26 − 4163.74 |  |  |
|  | 5 | | 5875 | 8273.04 | 4776.44 | −14676.51 − 26426.68 |  |  |
|  | 7 | | 7526 | 6880.66 | 3972.55 | −9566.69 − 24618.69 |  |  |
|  | 9 | | 15263 | 169.74 | 98 | 14841.34 − 15684.66 |  |  |
|  | 12 | | 15361 | 0 | 0 | 15361 − 15361 |  |  |
|  | 15 | | 15361 | 0 | 0 | 15361 − 15361 |  |  |
| MCP-1 (oral) | −2 | | 575.64 | 199.78 | 115.35 | 79.34 − 1071.93 | 0.024 |  |
|  | 1 | | 333.95 | 119.32 | 68.89 | 37.53 − 630.37 |  |  |
|  | 5 | | 901.36 | 292.81 | 169.05 | 173.97 − 1628.75 |  |  |
|  | 7 | | 673.1 | 215.09 | 124.18 | 138.78 − 1207.43 |  |  |
|  | 9 | | 746.02 | 86.02 | 49.67 | 532.33 − 959.71 |  |  |
|  | 12 | | 916.06 | 168.09 | 97.05 | 498.49 − 1333.63 |  |  |
|  | 15 | | 895.17 | 244.82 | 141.348 | 287.00 − 1503.34 |  |  |
| MIP-1β (intranasal) | −2 | | 69.61 | 31.79 | 18.35 | −9.37 − 148.58 | 0.005 |  |
|  | 1 | | 62.58 | 19.38 | 11.19 | 14.42 − 110.73 |  |  |
|  | 5 | 73.48 | | 27.3 | 15.76 | 5.67 − 141.29 |  | |
|  | 7 | 92.11 | | 38.51 | 22.24 | −3.57 − 187.78 |  |  |
|  | 9 | 90.5 | | 43.39 | 25.05 | −17.29 − 198.3 |  |  |
|  | 12 | 105.56 | | 43.66 | 25.21 | −2.91 − 214.03 |  |  |
|  | 15 | 117.06 | | 19.53 | 11.28 | 68.54 − 165.58 |  |  |

SD: standard deviation; SE: standard error; 95% CI: 95% confidence interval
